# Mir-142-3P regulates MAPK protein family by inhibiting 14-3-3η to enhance bone marrow mesenchymal stem cells osteogenesis

**DOI:** 10.1038/s41598-023-48950-4

**Published:** 2023-12-21

**Authors:** Ya-qian Liu, Yue-chen Xu, Zong-wen Shuai

**Affiliations:** 1 Department of Rheumatology and Immunology, The First Affiliated Hospital of Anhui Medical University, No. 218, Ji-Xi Road, Hefei, 230022 Anhui China; 2 Department of Radiotherapy, First Affiliated Hospital of Anhui Medical University, No. 218, Ji-Xi Road, Hefei, 230022 Anhui China

**Keywords:** Immunology, Rheumatology

## Abstract

Clinical studies have found *14-3-3η* to be associated with osteoporosis through undefined mechanisms. We aimed to investigate the role of *14-3-3η* in osteoporosis and its potential associations with miRNAs. The Gene Expression Omnibus(GEO) and Human Protein Atlas ^1^ databases were analyzed to examine both the mRNA and protein expression of *14-3-3η* in OP. Gene enrichment analyses were performed to explore the underlying mechanism of *14-3-3η* based on DAVID. miRWalk was used to predict the associated miRNAs. The statistics were analysed by R software and SPSS software. *14-3-3η* was overexpressed and knock down expressed in BMSCs by lentiviral vector transfecting. And BMSCs were induced by hypoxia. qRT-PCR and Western-Blot verified the expression of mRNA and protein. Scratch assay detected the migration of osteocytes. Co-immunoprecipitation and luciferase assay studied the 14-3-3η targeted protein and miRNA. overexpression and knock down of miRNA to verify the relationship of *14-3-3η* and target genes. The *14-3-3η* mRNA expression level was low in patients with osteoporosis, as corroborated by immunohistochemical staining images. Functional analyses revealed enrichment of the MAPK-associated cascade. *14-3-3η* was correlated with MAPK family proteins and five key miRNAs, including *mir-142-3p.* In addition, *14-3-3η* knockdown in BMSCs increased the mRNA and protein expression levels of Hif-α, VEGF, BMP-2, OPN, OST, and Runx2, and enhanced the cells migration ability. Under hypoxic conditions, Hif-α and BMP-2 protein expression levels were upregulated, whereas those of 14-3-3η and MAPK3 were downregulated. Co-immunoprecipitation experiments showed decreased binding of 14-3-3η to MAPK3. *14-3-3η* knockdown produced the same results as hypoxia induction. Adding caspase3 inhibitor and knocking down *14-3-3η* again prevented MAPK3 cleavage by caspase3 and inhibited BMP-2 expression. Moreover, under hypoxic conditions, *miR-142-3P* expression was upregulated and luciferase assays revealed *14-3-3η* as its target gene. *miR-142-3P* overexpression decreased mRNA and protein levels of 14-3-3η and MAPK3, while increasing BMP-2 expression. *miR-142-3P* knockdown reversed these results. BMSC osteogenesis was suppressed by *14-3-3η*, whereas *miRNA-142-3p* promoted it through the inhibition of *14-3-3η*.

## Introduction

Osteoporosis is a systemic bone metabolic disease that can easily lead to fractures and other serious complications^1^. Regulating the proliferation, migration, and osteogenic differentiation of bone marrow mesenchymal stem cells (BMSCs) can promote bone formation, improve bone damage, and treat various kinds of osteoporosis, such as primary osteoporosis, rheumatoid arthritis(RA)-induced secondary osteoporosis, hormone osteoporosis, and others. *14-3-3η* is an independent risk factor for RA-induced osteoporosis (*OR*, 1.503; 95% *CI*, 1.116–2.025;* P* < 0.01), with mean levels (3.26 ng/mL) being significantly higher than in control groups of patients with other diseases, patients without RA who had osteoporosis, and healthy controls (*P* < 0.01)^2^. A negative association between *14-3-3η* and osteoporosis has also been reported^3^, but the mechanisms underlying this association remain unclear, despite the report of a potential 14-3-3 interaction site on the bone regulator protein schnurri-3^4^.

*14-3-3η* binds and regulates at least 200 kinds of molecules with a wide range of biological functions^5^. MAPK signaling pathway regulates many cell processes, such as cell division, differentiation, release of inflammatory mediators^6^, and inhibit osteoclastogenesis^7^. Jamie et al. demonstrated that Bioactive silica nanoparticles target autophagy, NF-κB, and MAPK pathways to prevent conversion to osteoclasts^7^. In view of these extensive biological functions and the association with osteoporosis, we used bioinformatics methods to investigate the hypothesis that *14-3-3η* can influence the MAPK family and osteogenesis; we found a strong association of *14-3-3η* with other MAPK proteins in patients with primary osteoporosis, and carried out biogenic verification experiments to assess the effect of *14-3-3η* expression on the expression of key MAPK protein and osteogenic-related proteins. Meanwhile, Studies suggest that miRNAs play crucial roles bone metastasis by regulating osteogenesis or osteoclast differentiation and remodeling of the bone microenvironment^8^.For instance, *miRNA-133a* is involved in the regulation of postmenopausal osteoporosis through promoting osteoclast differentiation^9^. *miRNA-483-5p* is also involved in the pathogenesis of osteoporosis by promoting osteoclast differentiation^10^, *miRNA-429* suppresses osteogenic differentiation of human adipose-derived mesenchymal stem cells under oxidative stress via targeting SCD-1^11^, *miR‑142‑3p* promotes osteoblast differentiation by modulating Wnt signaling^12^. Therefore, we additionally assessed associations between miRNAs and 14-3-3η proteins, which are thought to be involved in the osteogenic differentiation of bone marrow mesenchymal stem cells (BMSCs).

## Materials and methods

### Data collection and analysis

GSE37745 database data was downloaded from GEO^13^ using the GEO query package of R (4.0.2), including total RNA data of BMSC samples from four healthy donors and five patients with osteoporosis. The probes with the largest signal value were retained when encountering probes corresponding to the same molecule. The differential expression between samples analyzed using the Limma package and the results were visualized with the ggplot2 package. The HPA database^14^ contains immunohistochemistry (IHC) data of *14-3-3η* from bone marrow and soft tissue types.

### Gene enrichment analysis based on 14-3-3η-coexpressed genes

The COEXPEDIA^15^ bioinformatics tool was used to examine the co-expressed genes of *14-3-3η* with the GO and KEGG pathway enrichment tool DAVID^16^. Then we used R software with the cluster Profiler and ggplot2 packages to analyze and visualize the correlations between multiple key genes and *14-3-3η*.

### Target miRNA prediction

*14-3-3η*-related miRNAs were identified by using miRWalk^17^, a web interface with precise tools for predicting miRNA-target interactions.

### Cell cultures

BMSCs were prepared by the Cellcook Company (Guangzhou, Guangdong, China). They were derived from large male dog (12–16 kg, 6–8 weeks old) iliac marrows provided by Laboratory Animal Center of Anhui Medical University. Bone marrow puncture was performed at the iliac crest and 5 mL of marrow were extracted and mixed with 5 mL of Dulbecco's modified Eagle media (DMEM). The mixture was centrifuged at 1000 r/ min for 8 min and the precipitate was retained. The cells were resuspended in 15 ml DMEM supplemented with 10% (v/v) fetal bovine serum (FBS; Biological Industries, Israel), 1% (w/v) penicillin (Invitrogen, Carlsbad, CA, USA), and 1% (w/v) sodium pyruvate (Invitrogen), and then transferred into a cell culture flask. The cells were incubated at 37 °C with 5% CO_2_ for 48 h until adherent growth. Once the cells reached 90% confluence, they were digested with 0.25% (v/v) trypsin and subcultured serially. BMSCs grew adhering to the flask wall and displayed a fusiform or polypseudopod fibroblast morphology. The analysis of identification was performed with a flow cytometer (BD Biosciences, Franklin Lakes, NJ, USA) using CD29 and CD34 as specific stem cell markers (BMSCs are CD29-positive and CD34-negative).

Our Ethics Committee approved this study. Written informed consents were obtained from all data donors.

### Hypoxia induction in BMSCs

We placed the cell culture plates in an airtight container and exposed the cells to hypoxic conditions at specific timepoints. A mixed gas of 95% N_2_ and 5% CO_2_ was charged through the container for 30 min and sealed the air inlet/outlet. BMSCs were placed into separate hypoxia boxes during the intervention.

### Lentivirus transductions

Sh14-3-3η lentiviral particles or control lentiviral particles were prepared by the Sigma Company (St. Louis, MO, USA). For lentiviral infections, we incubated BMSCs with lentiviral particles with 8 μg/mL of polybrene for 24 h. Following the incubation, we replaced the infection medium with fresh culture medium.

### RNA extraction and qRT-PCR

The total RNA were isolated from cells using TRIzol™ reagent (Invitrogen). After reverse transcription into cDNA with a reverse transcription kit (Takara, Kusatsu, Shiga, Japan), we set up qRT-PCR reactions using TB Green®Premix Ex Taq™ (Takara) according to the manufacturer’s instructions. The *14-3-3η* gene-specific primers and *miR-142-3P* were synthesized by Sangon (Shanghai, China). qRT-PCR cycling conditions included an initial denaturing step (94 °C for 5 min), a denaturation extension (30 cycles at 94 °C for 40 s, followed by 57 °C for 50 s), an annealing step (72 °C for 1 min), and an extension step (72 °C for 5 min). We used the expression of beta-actin as an internal reference to normalize expressions of *14-3-3η*. We carried out the qRT-PCR reactions in an ABI Step One Plus instrument (Applied Biosystems, Foster City, CA, USA). All the primer sequences are listed in the Supplementary Table 1 section.

### Western blot analysis

After culturing infected BMSCs in EGM, we lysed them using RIPA lysis buffer containing 1% (w/v) PMSF (Biosharp, Hefei, Anhui, China). We then denatured the proteins with 4% SDS. We measured protein concentrations using a Coomassie brilliant blue staining, separated the protein samples on SDS- PAGE, and transferred them onto PVF membranes (Invitrogen). After blocking the membranes with 5% (w/v) skimmed milk, we probed them with various antibodies overnight at 4 °C. We used primary antibodies specific for β-catenin, 14-3-3η, bone morphogenetic protein-2 (BMP-2), osteopontin (OPN), osteocalcin (OST), runt-related transcription factor 2 (RUNX2), hypoxia inducible factor-α (Hif-α), and vascular endothelial growth factor (VEGF). Subsequently, we incubated the membranes with a secondary antibody (Zsbio, Beijing, China) for 1 h and visualized the bands using a chemiluminescent substrate ECL kit (Millipore, Billerica, MA, USA). Finally, we detected and quantified the proteins using a ChemiDoc-XRS + apparatus (Bio-Rad, Richmond, CA, USA).

### Co-Immunoprecipitation (Co-IP) experiments

We added 500 µL PBS and protein A/G beads to 1.5 mL transferring tubes. We mixed the tubes with 14-3-3η, MAPK3, or control antibody, and placed them on a low-speed rotating shaker for 2–3 h at room temperature. Next, we centrifuged the tubes at 14,000 g 4 °C for 15 min and transferred the supernatants to the new tubes. We then washed the protein A/G-agarose beads twice with PBS and prepared a 50% protein A/G agarose working solution (in PBS). We added 100 μL of the solution to each 1-mL sample and shook them on a horizontal shaker for 10 min. We centrifuged the tubes at 14,000 g 4 °C for 15 min and transferred the supernatants to new tubes. The protein A/G agarose beads were washed 2–3 times. We quantified the total protein in each sample and diluted it to 1 μg/μL with PBS to decrease the detergent concentration. We then added buffer and lysed cells at 4℃ for 1 h and centrifuged the samples at 14,000 rpm for 15 min, before mixing the supernatants with protein A/G beads coupled with 14-3-3η, MAPK3, or control antibody. We added appropriate amounts of primary antibody and incubated the antigen–antibody complex samples on a rotating shaker at 4 °C overnight. The next day, we centrifuged the samples at 14,000 g for 3–5 s, collected the pellets, and washed them with pre-chilled washing buffer three times. We collected the supernatants and heated them at 95 °C for 8 min before proceeding to SDS-PAGE.

### Dual-luciferase reporter assay

We designed and amplified the 14-3-3η3′-UTR sequence in Plko.1 plasmids by PCR and sub-cloned it into the psiCheck2.0 vector for luciferase reporter assays and named the resulting constructs psi-14-3-3η3′-UTR-WT and psi-14-3-3η3′-UTR-Mut. Briefly, we incubated 293 T cells in 24-well plates and co-transfected them with 100 ng of psi-check 2.0 luciferase vectors containing the wild-type or mutated 14-3-3η3′-UTR, and *miR-142-3P* mimics/inhibitor or normal control according to the experimental groups. We performed dual-luciferase reporter assays (Promega, Madison, WI) according to the manufacturer's instructions. We performed the transfection experiments in triplicate.

### Wound healing assays

First, BMSCs were spreaded with a cell density of 5–10 × 10^5^/mL on 24-well plates (500 μL per well) and incubated them with DMEN medium containing 10% FBS to form cell monolayers. Then transfected the cells with 50 nM sh14-3-3η (Ribobio, Guangzhou, China). After 24 h, we used a 200-µL sterile plastic micropipette tip to scratch the cell monolayer drawing wound lines; then washed the cell layer with PBS three times and cultured parallel samples in DMEM with 2% FBS medium. After 0, 12, and 24 h of incubation at 37 °C with 5% CO_2_, Cell migration rates were calculated by photographing the cell monolayer wounds.

### Statistical analyses

Statistical analyses were conducted by SPSS 17.0 and R 4.0.2 software. All experiments were carried out in triplicates, and the results were represented as means ± SDs. Comparisons between two groups were analyzed by Student’s T-tests. One-way analysis of variance or two-way analysis of variance was used for comparisons among multiple groups. Diferences were considered statistically signifcant when *P* < 0.05.

### Ethical approval and consent to participate

All animals were kept in a pathogen-free environment and fed ad lib. The procedures for care and use of animals were approved by the Ethics Committee of the Animal Welfare & Ethics Committee of Anhui Medical University and all applicable institutional and governmental regulations concerning the ethical use of animals were followed. All procedures were conducted in accordance with the “Guiding Principles in the Care and Use of Animals. All procedures were conducted in accordance with the ARRIVE guidelines.

### Consent for publication

Informed consent was obtained from all individual participants included in the study.

## Results

### 14-3-3η was downregulated in primary osteoporosis

**Figure 1:**
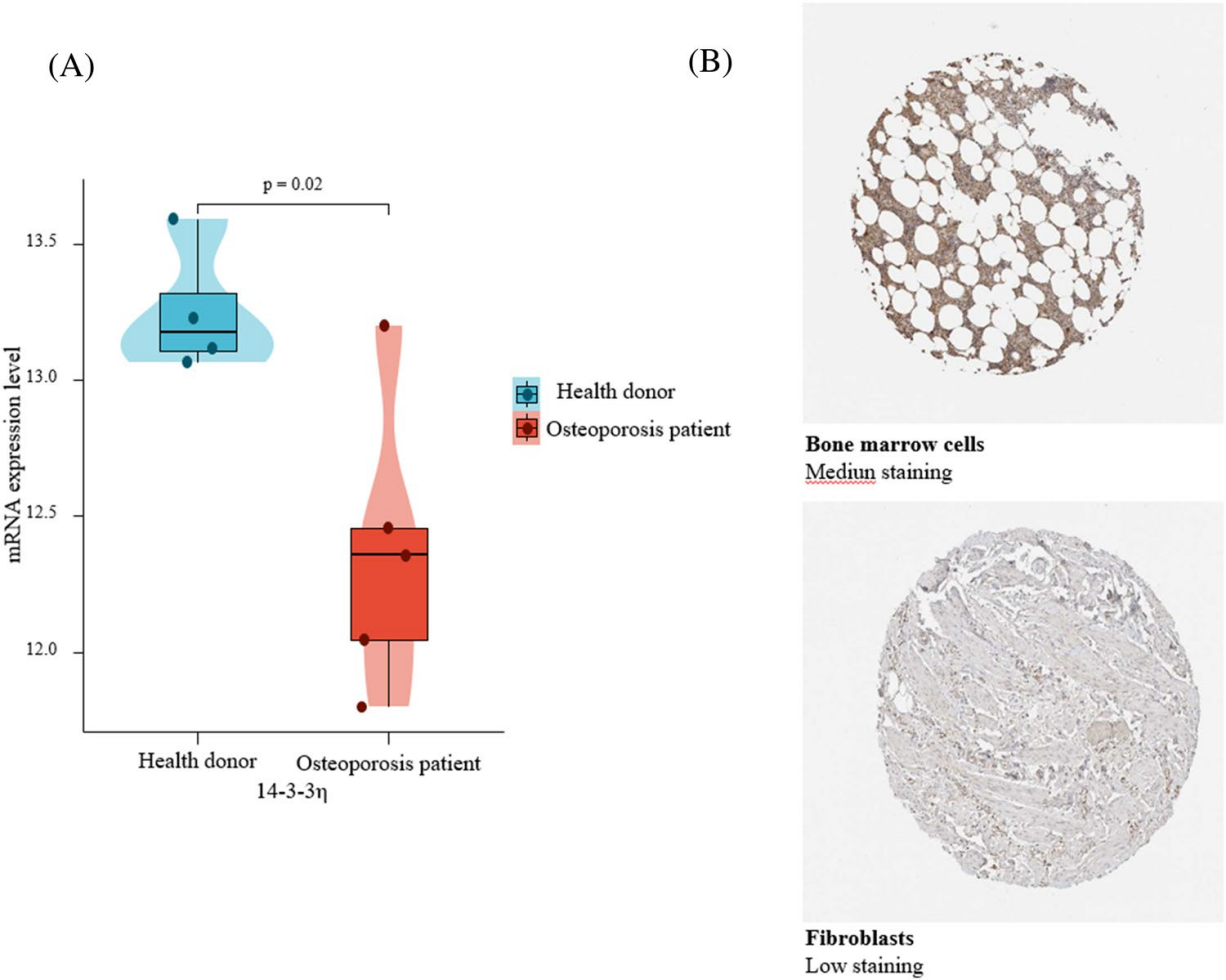
14-3-3η expression levels. (**A**) Differential *14-3-3η* mRNA expression between patients with osteoporosis and healthy donors in the GSE37745 database. Y-axis: Transcripts per kilobase of exon model per million mapped reads. (**B**) Immunohistochemistry staining of 14-3-3η protein in fibroblasts and bone marrow cells in the HPA database.

We analyzed *14-3-3η* mRNA expression in patients with primary osteoporosis and health donor RNA sequencing data from the GEO database (GSE35958 dataset). The results revealed that the *14-3-3η* mRNA expression level was lower in patients with osteoporosis than in healthy donors (*P* < 0.05, Fig. 1A). Moreover, IHC staining data from the HPA database demonstrated low levels of *14-3-3η* expression in fibroblasts and moderate levels of expression in bone marrow cells (Fig. 1B). These results indicate that the 14-3-3η mRNA and protein levels were lower in samples of patients with osteoporosis than in healthy donor samples.

### Functional annotation and pathway enrichment of 14-3-3η-coexpressed genes and prediction of 14-3-3η-associated genes

**Figure 2:**
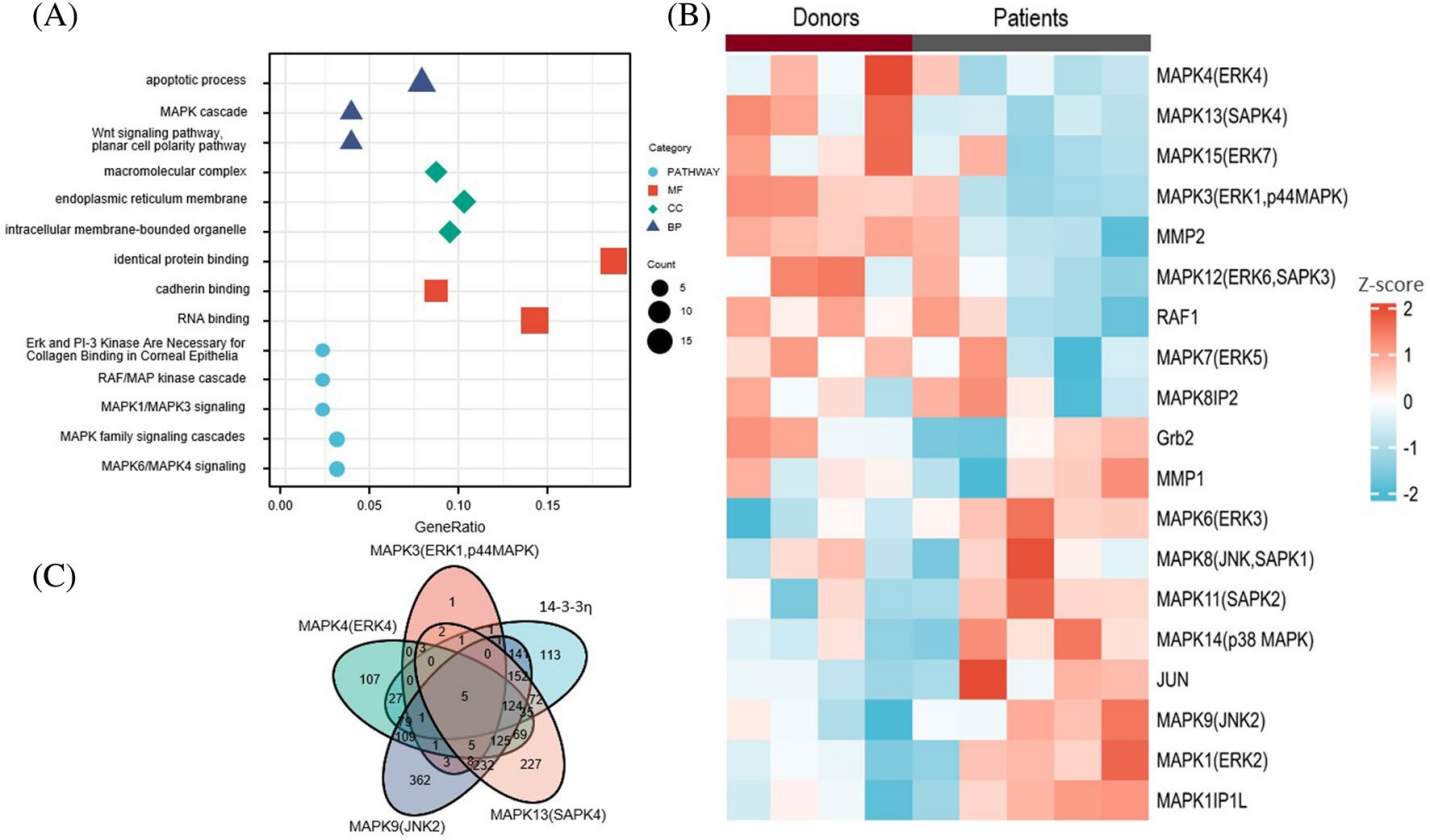
Gene enrichment analysis for *14-3-3η*. (**A**) Enriched GO terms and KEGG pathways. (**B**) Heatmaps showing the correlation between the MAPK gene family and *14-3-3η* in the GSE37745 database. The left four columns represent donors and the five right columns represent patients. (**C**) The predicted target miRNAs are presented in a Venn diagram, with only the intersections selected to increase specificity.

The KEGG pathway enrichment analysis of the *14-3-3η*-coexpressed genes revealed enrichment in MAPK, RAF/MAP kinase, MAPK1/MAPK3, MAPK family, and MAPK6/MAPK4 signaling pathways (Fig. 2A), as well as macromolecular complex, endoplasmic reticulum membrane, and intracellular membrane bounded organelles (cellular component terms). In addition, we found involvement in identical protein binding, cadherin binding, and RNA binding (molecular function terms).

The enriched GO terms and KEGG pathway analysis results displayed potential *14-3-3η* functions, allowing us to further explore the correlations between 14-3-3η and multiple key genes. Figure 2B illustrates the association between *14-3-3η* and some key MAPK signaling pathway proteins in the database (left four columns show donors’ results and five right columns show patients’ results). We observed significant correlations between the *14-3-3η* expression levels and many MAPK family proteins and downstream genes, including MAPK1 (ERK2), MAPK3 (ERK, p44MAPK), MAPK4 (ERK4), MAPK6 (ERK3), MAPK7 (ERK5), MAPK8 (JNK, SAPK1), MAPK9(JNK2), MAPK11 (SAPK2), MAPK12 (ERK6, SAPK3), MAPK13 (SAPK4), MAPK15 (ERK7), MAPK8IP2, *JUN*, *RAF1*, *MMP1*, *MMP2*, and *Grb2.*

In the correlation analysis of multiple genes, we found MAPK3, MAPK4, MAPK9, and MAPK13 to be correlated with *14-3-3η* with good consistency. MIRWALK bioinformatics predicted miRNAs associated with MAPK3, MAPK4, MAPK9, MAPK13, and 14-3-3*η*, and Fig. 2C shows the intersection. Finally, we identified five key miRNAs, namely *miR-142-3p*, *miR-27b-5p*, *miR-196a-5p*, *miR-99a-3p*, and *miR-198*.

### 14-3-3η knockdown in BMSCs

**Figure 3:**
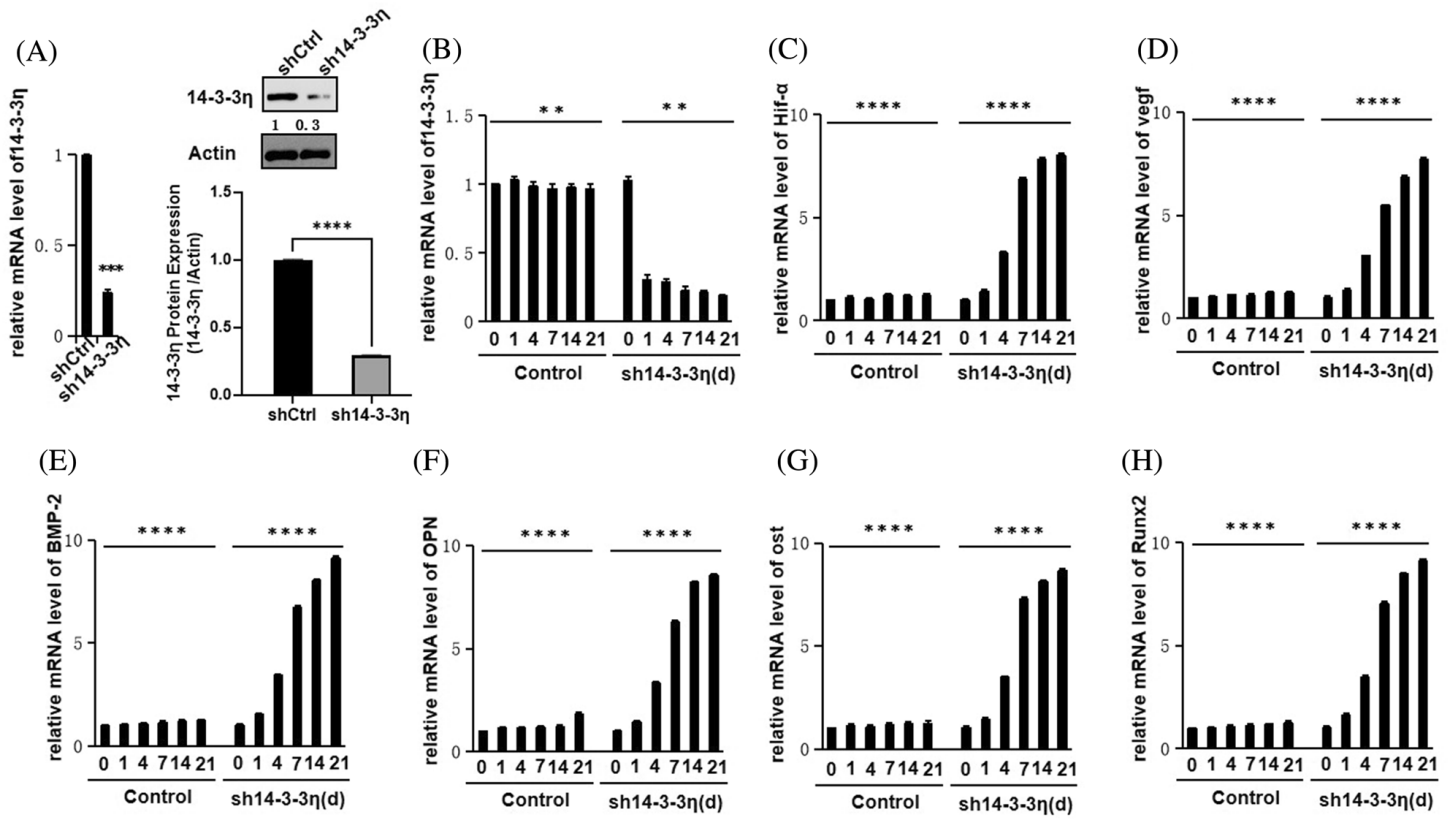
Expression of osteogenic factors after *14-3-3η* knockdown. (**A**) *14-3-3η* mRNA and protein expression levels in BMSCs after *14-3-3η* knockdown. (**B**) *14-3-3η* mRNA expression levels in control and *14-3-3η* knockdown cells on days 0, 1, 4, 7, 14, and 21. (**C**–**H**) Hif-α, VEGF, BMP-2, OPN, OST, and *Runx2* mRNA expression levels in control and *14-3-3η* knockdown cells on days 0, 1, 4, 7, 14, and 21.

After lentiviral infection, the *14-3-3η* mRNA and protein expression levels were both significantly decreased (Fig. 3A).

### qRT-PCR mRNA expression of osteogenic factors after 14-3-3η knockdown

The mRNA levels of osteogenic factors (Hif-α, VEGF, BMP-2, OPN, OST and Runx2) were upregulated after *14-3-3η* knockdown. The *14-3-3η* mRNA expression levels were decreased in the sh14-4-4η-transfected cells on days 0, 1, 4, 7, 14, and 21, and they were normal in control cells (Fig. 3B). As shown in Fig. 3C–H, the mRNA expression levels of osteogenic factors were higher in sh14-4-4η-transfected cells than in control cells on days 0, 1, 4, 7, 14, and 21 (*P* < 0.05). With time, the expression levels of osteogenic factors displayed an increasing trend (*P* < 0.05).

### Western blot protein expression levels of osteogenic factors after 14-3-3η knockdown

**Figure 4:**
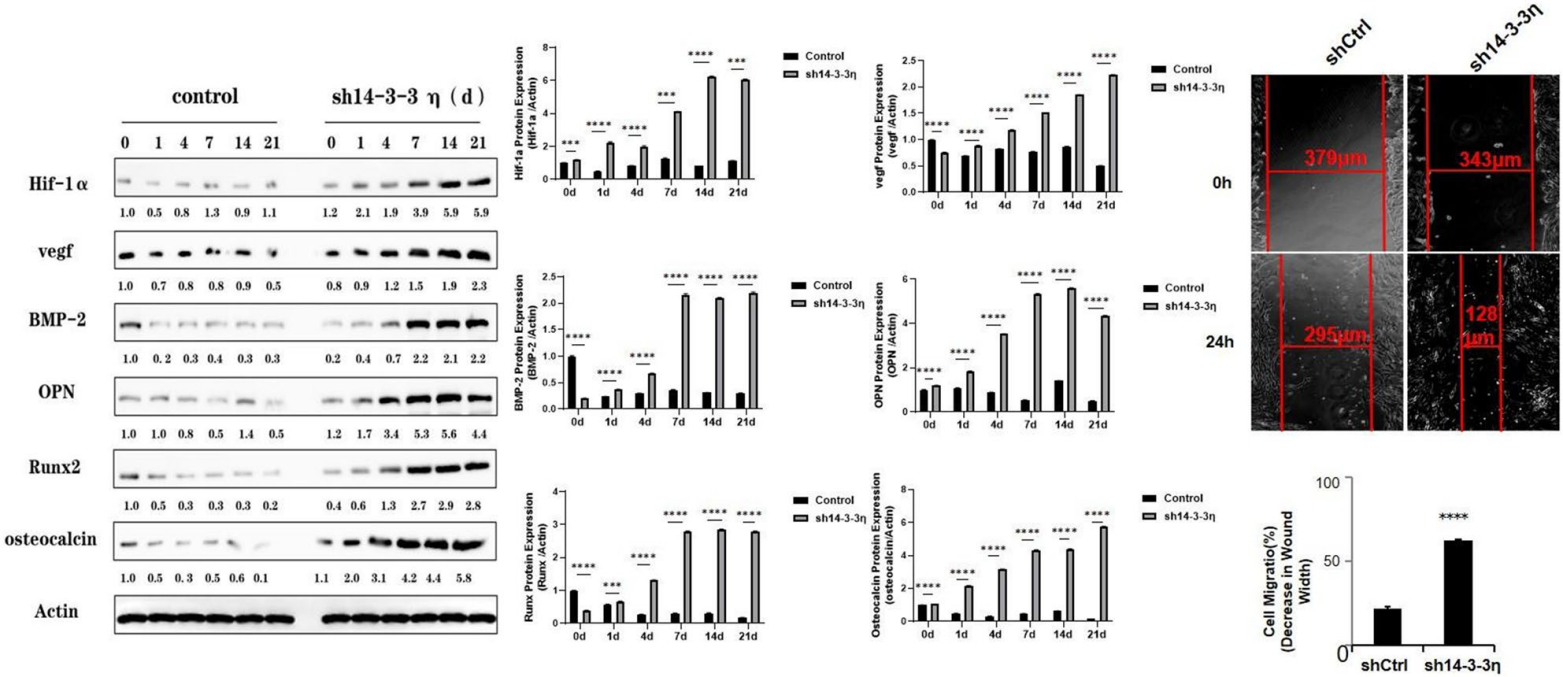
Protein expression levels of osteogenic factors and scratch assay after *14-3-3η* knockdown. (**A**) Hif-α, VEGF, BMP-2, OPN, OST, and Runx2 protein levels in control and *14-3-3η* knockdown cells on days 0, 1, 4, 7, 14, and 21. (**B**) BMSC migration and proliferation after *14-3-3η* knockdown.

Consistently with our mRNA results, the protein expression levels of Hif-α, VEGF, BMP-2, OPN, OST, and Runx2 were significantly higher (*P* < 0.05) in the sh14-3-3η-transfected cells than in control cells on days 0, 1, 4, 7, 14, and 21 (Fig. 4A).

### Scratch assay of 14-3-3η-knockdown BMSCs

After counting the control group and sh14-3-3η group BMSCs, we transferred equivalent numbers of BMSCs to 12-well plates. Once the cells were completely adherent to the flask wall, we detected their migration ability using scratch assays. The migrating sh14-3-3η cells were significantly faster than the control cells (Fig. 4B). These results show that *14-3-3η* knockdown promoted BMSC migration.

### Expression levels of 14-3-3η, MAPK3, and osteogenic factors upon hypoxia induction

**Figure 5:**
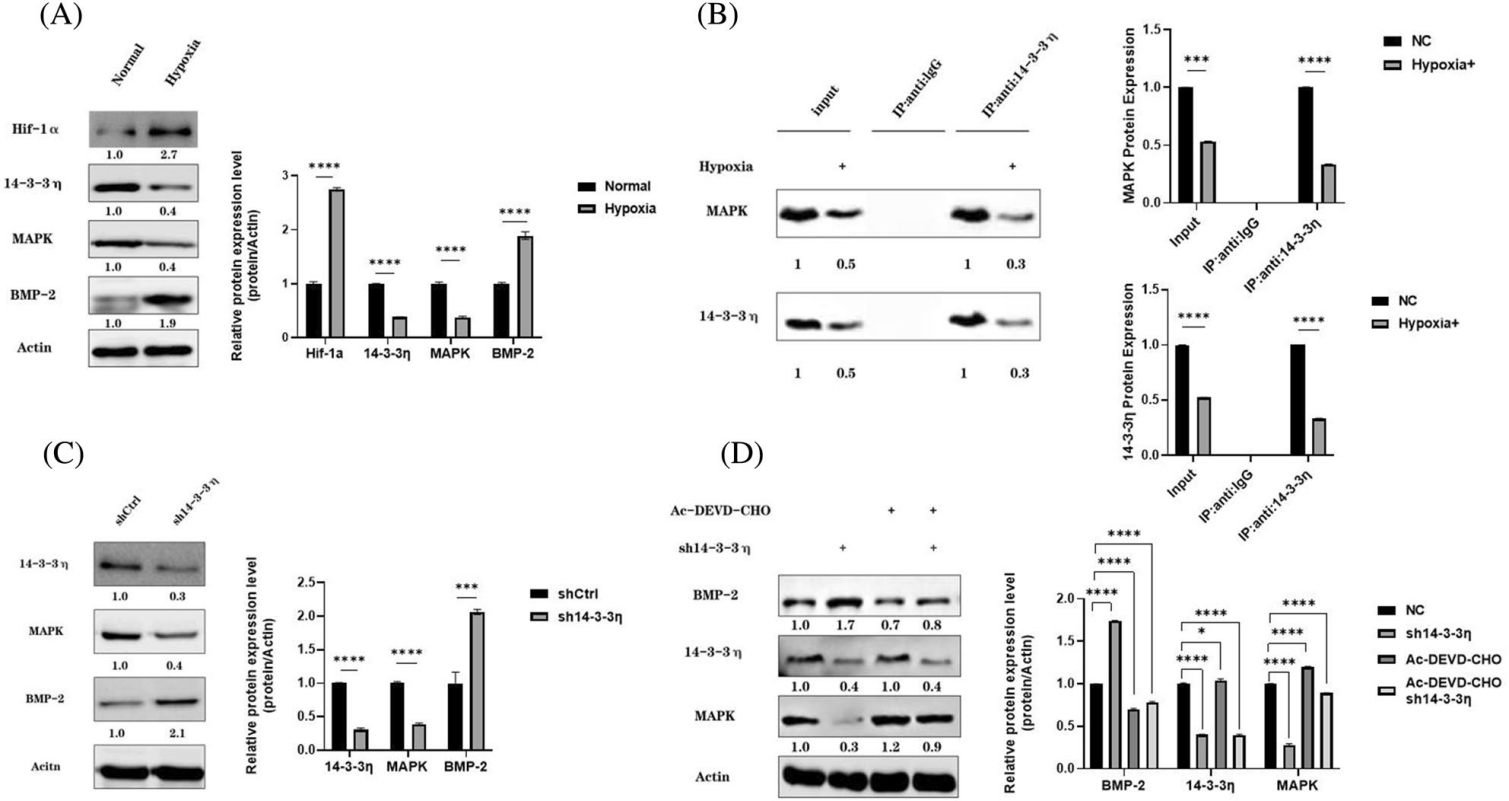
Associations between miR-142-3P, 14-3-3η, MAPK3 and osteogenic factors under hypoxic conditions. (**A**) 14-3-3η, MAPK3, Hif-1a, and BMP-2 expression levels. (**B**) Co-immunoprecipitation of 14-3-3η with MAPK3. (**C**) MAPK3 and BMP-2 expression after *14-3-3η* knockdown. (**D**) Expression of MAPK3 and BMP-2 in *14-3-3η* knockdown cells after caspase3 inhibitor addition.

Under hypoxic conditions, our western blot results show that the 14-3-3η and MAPK3 protein expression levels were downregulated, whereas those of Hif-α and BMP-2 were upregulated in BMSCs (*P* < 0.05), compared with the levels in the control cells (Fig. 5A).

The results of the co-immunoprecipitation experiments show that the binding of 14-3-3η to MAPK3 decreased under hypoxic conditions (Fig. 5B).

To verify the association between the 14-3-3η and MAPK3 under hypoxic conditions, we knocked down *14-3-3η* in BMSCs and found that the MAPK3 protein expression was downregulated, whereas that of osteogenic factor BMP-2 was upregulated (Fig. 5C).

Moreover, we treated *14-3-3η* knockdown BMSCs with the caspase3 inhibitor AC-DEVD-CHO and found that the MAPK3 protein expression was not downregulated. The MAPK3 protein was not cleaved by caspase3 due to the protection provided by AC-DEVD-CHO (Fig. 5D), and the BMP-2 expression was not upregulated.

Thus, *14-3-3η* knockdown resulted in downregulated MAPK3 protein expression and increased osteogenic factors expression. However, when caspase3 inhibitor was added to protect the MAPK3 protein from cleavage by Caspase 3, the MAPK3 protein expression did not decrease and the osteogenesis expression did not either. This suggests that *14-3-3η* may act like a caspase 3 inhibitor during the MAPK3 downregulation of osteogenic factors expression.

### Expression levels of 14-3-3η and miR-142-3P upon hypoxia induction

**Figure 6:**
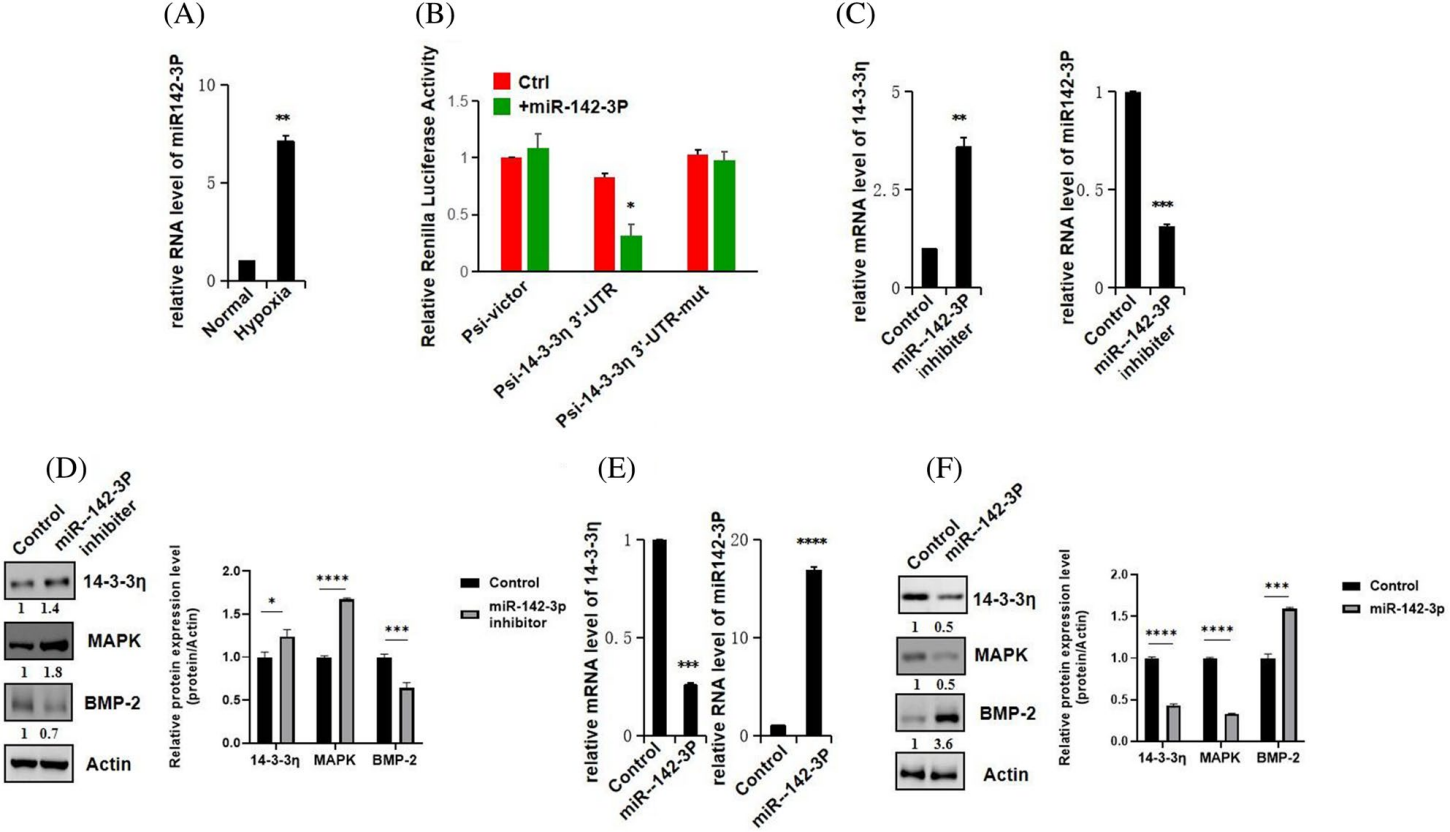
Associations of miR-142-3P, 14-3-3η, MAPK3, and osteogenic factors under hypoxic conditions. (**A**) *miR-142-3P* mRNA levels in control and hypoxia groups. (**B**) Luciferase activity among cells expressing the Psi-14–3-3n 3'-UTR-mut plasmid. (**C**) *14-3-3η* and *miR-142-3P* mRNA expression levels under miR-142-3P. (**D**) Protein expression levels of 14-3-3η, MAPK3, and BMP-2 under *miR-142-3P* knockdown. (**E**) *14-3-3η* and *miR-142-3P* mRNA expression levels under *miR-142-3P* overexpression. (**F**) 14-3-3η, MAPK3, and BMP-2 protein expression levels under *miR-142-3P* overexpression.

Under hypoxic conditions, the protein expression of levels of 14-3-3η and MAPK3 were downregulated, whereas the protein expression levels of osteogenesis markers were upregulated. However, under hypoxic conditions, the miR-142-3P levels were significantly higher (*P* < 0.05) than those under normal conditions (Fig. 6A).

### Luciferase assays in cells transfected with Psi-14-3-3η3’-UTR-mut plasmid

To investigate the regulatory interaction between *miR-142-3P* and *14-3-3η*, we constructed wild-type and mutant luciferase reporter vectors, as show in Fig. 6B. The relative luciferase activity (△Ct) of cells transfected with either pmirGLO-mut-AKT or Psi-vector plasmids showed similar luciferase activities (*P* > 0.05). The cells transfected with psi-14-3-3η 3′-UTR vector plasmid displayed lower luciferase activity than the control cells (*P* < 0.005). However, the luciferase activities of the cells transfected with psi-14-3-3η 3′-UTR and those transfected with blank Psi-Victor plasmid differed significantly (*P* > 0.05). Our results suggest that *14-3-3η* is the target gene of *miR-142-3P*, and that *miR-142-3P* binds to the 3′-UTR region of *14-3-3η*. Thus, *14-3-3η* seems to be inhibited through post-transcriptional regulation.

### Expression levels of MAPK3, BMP-2, and 14-3-3η after miR-142-3P knockdown

Our qRT-PCR results in cells with *miR-142-3P* knockdown show that the expression levels of *miR-142-3P* were lower and those of 14-3-3η were higher than the levels in the control cells (Fig. 6C). Moreover, western blot analysis after *miR-142-3P* knockdown showed that the protein expression levels of 14-3-3η and MAPK3 were significantly higher and those of osteogenic protein BMP-2 were lower than those in control cells (Fig. 6D).

### MAPK, BMP-2, and 14-3-3η mRNA expression levels in cells overexpressing miR-142-3P

Our qRT-PCR results show that *miR-142-3P* overexpression led to high *miR-142-3P* and low *14-3-3η* expression levels (Fig. 6E). In addition, our western blot experiments with cells overexpressing *miR-142-3P* showed significantly lower 14-3-3η and MAPK3 protein levels and higher osteogenic protein BMP-2 levels than in control cells (Fig. 6F).

## Discussion

Osteoporosis, a common complication of rheumatological diseases due to long-term systemic inflammation and corticosteroid use, significantly impairs patients’ quality of life. Clinical observations have revealed a correlation between *14-3-3η* and osteoporosis. Zeng T et al^2^. reported that serum 14-3-3η levels were higher in 113 newly diagnosed patients with RA who had never used disease-modifying antirheumatic drugs than in 162 patients without RA who presented arthritis or autoimmune diseases. Logistic regression analysis identified *14-3-3η* as an independent risk factor for RA-related osteoporosis. Therefore, *14-3-3η* may be a promising biomarker for disease monitoring in patients with RA. A previous study by our team also evaluated serum levels of 14-3-3η by enzyme-linked immunosorbent assays in patients with early RA (259 patients and 80 healthy controls)^3^ and a linear correlation analysis showed that the serum 14-3-3η protein level was negatively correlated with the bone mineral density in the lumbar spine and femur (*P* < 0.0001). Moreover, the serum 14-3-3η protein levels among patients with normal bone mass (2.73/3.79), osteopenia (3.15/4.86), and osteoporosis (6.34/6.42) were different (*χ*^2^ = 7.974, *P* < 0.05); and, therefore, the serum 14-3-3η protein level was associated with the bone mass. However, to the best of our knowledge, only one study has explored the possible mechanisms of the role of the 14-3-3 protein family in osteoporosis: In silico analysis of the protein sequence of mammalian schnurri-3, a suppressor of mouse bone formation and candidate target for novel osteoporosis therapeutics^4^, identified potential *14-3-3σ* interaction sites. The study found that 14-3-3η expression was lower at the transcriptional and proteomic levels in osteoporosis than in control samples. Knocking down *14-3-3η* in BMSCs upregulated protein and mRNA expression of osteogenic indicators. In addition, a wound healing assay showed that knocking down *14-3-3η* enhanced the proliferation and migration of BMSCs.

KEGG pathway results show that the *14-3-3η* co-expressed genes were mainly enriched in BPs of the MAPK-associated pathway. In addition, *14-3-3η* was significantly correlated with many MAPK family protein and downstream genes of the GSE35958 database. Studies have shown evidence for *14-3-3* family members activating JAK-STAT, MAPK/ERK, and other signaling pathways, and inducing interactions with ASK-1, PKC, MMP, RAF-1, TNF-α, and other inflammatory factors^18,19^. Our initial results with the osteoporosis database were consistent with those results. Therefore, we exposed BMSCs to hypoxia to induce osteogenesis, and found that osteogenic factor expression was enhanced, whereas the 14-3-3η and MAPK3 expressions were downregulated, probably due to the reduced binding of 14-3-3η with MAPK3 under hypoxic conditions, a phenomenon predicted to protect MAPK kinase from caspase3 cleavage^20^. Therefore, after adding caspase3 inhibitor and knocking down *14-3-3η* again under hypoxic conditions, we observed that MAPK3 was not cleaved by caspase3 and BMP-2 expression was not upregulated. Moreover, co-immunoprecipitation experiment results showed a decreased binding of 14-3-3η to MAPK3. This indicates that the interaction between 14-3-3η and MAPK may be an important mechanism to inhibit the osteogenic differentiation of BMSCs.

miRNAs plays important regulatory roles during osteogenesis, BMSC differentiation, and inflammatory immunity^21^. We predicted 5 miRNA associations with 14-3-3η and MAPK3 through bioinformatics, and our luciferase assays indicate that *14-3-3η* is the target gene of *miR-142-3P*. Previous studies have demonstrated that miRNA profiling of monocyte-to-osteoclast differentiation identified *miR-142-3p* as a miRNA that is significantly, differentially expressed throughout osteoclastogenesis^22^. Kim et al. reported that *miR-142-3p* is involved in TGF-β3-mediated region-dependent chondrogenesis through regulation of ADAM9^23^. Enforced expression of *miR-142-3p* via transient transfection with *miR-142-3p* mimic inhibited cell-to-cell contact and fusion, decreased protein kinase C alpha expression, and ultimately reduced cell viability. *miR-142-3p* was also identified as significantly differentially expressed during monocyte-to-macrophage differentiation and overexpression of *miR-142-3p* prevented their conversion to osteoclasts. Furthermore, the inhibitory effect of *miR-142-3p* on osteoclastogenesis extended to the conversion of a third osteoclast precursor cell type- dendritic cells^24^. Moreover, *miR-142-3P* knockdown upregulated the expressions of 14-3-3η and MAPK3 to inhibit osteogenesis of BMSCs, and *miR-142-3P* overexpression reversed these results. On the one hand, *miR-142-3P* is likely to form a negative feedback loop through the 14-3-3η and MAPK pathways to promote osteogenic differentiation of BMSCs and may target the MAPK signaling pathway inhibitor, thereby promoting osteoblast differentiation. On the other hand, *miR-142-3P* may be involved in long-term chronic inflammation, stimulating local osteogenesis, inducing inflammatory transcription factors, and osteogenesis factors, such as BMP-2 (Supplementary information 1).

## Conclusion

Our results demonstrate that *miR-142-3P* regulates MAPK3, a key MAPK signaling pathway protein, by targeting *14-3-3η* to inhibit BMSC osteogenesis. This finding improves our understanding of the role of 14-3-3η in BMSC osteogenesis and may aid the development of diagnostic and therapeutic strategies for osteoporosis. Future studies should evaluate the role of *miR-142-3P* and *14-3-3η* in BMSCs using a large sample size.

### Supplementary Information

Supplementary Figures.

Supplementary Table 1.

## Data Availability

The GEO database that support the findings of this study are openly available in: GEO Accession viewer (nih.gov), GEO accession:GSE37745. All data generated or analyzed during this study are included in this published article.
