# Supplementary material for: Mir-142-3P regulates MAPK protein family by inhibiting 14-3-3η to enhance bone marrow mesenchymal stem cells osteogenesis
Source: Sci Rep. 2023 Dec 21;13:22862. doi: 10.1038/s41598-023-48950-4 (PMC10739902; doi:10.1038/s41598-023-48950-4)
Supplement: Supplementary file 1 — Supplementary Figures. [file 41598_2023_48950_MOESM1_ESM.doc]

Fig3


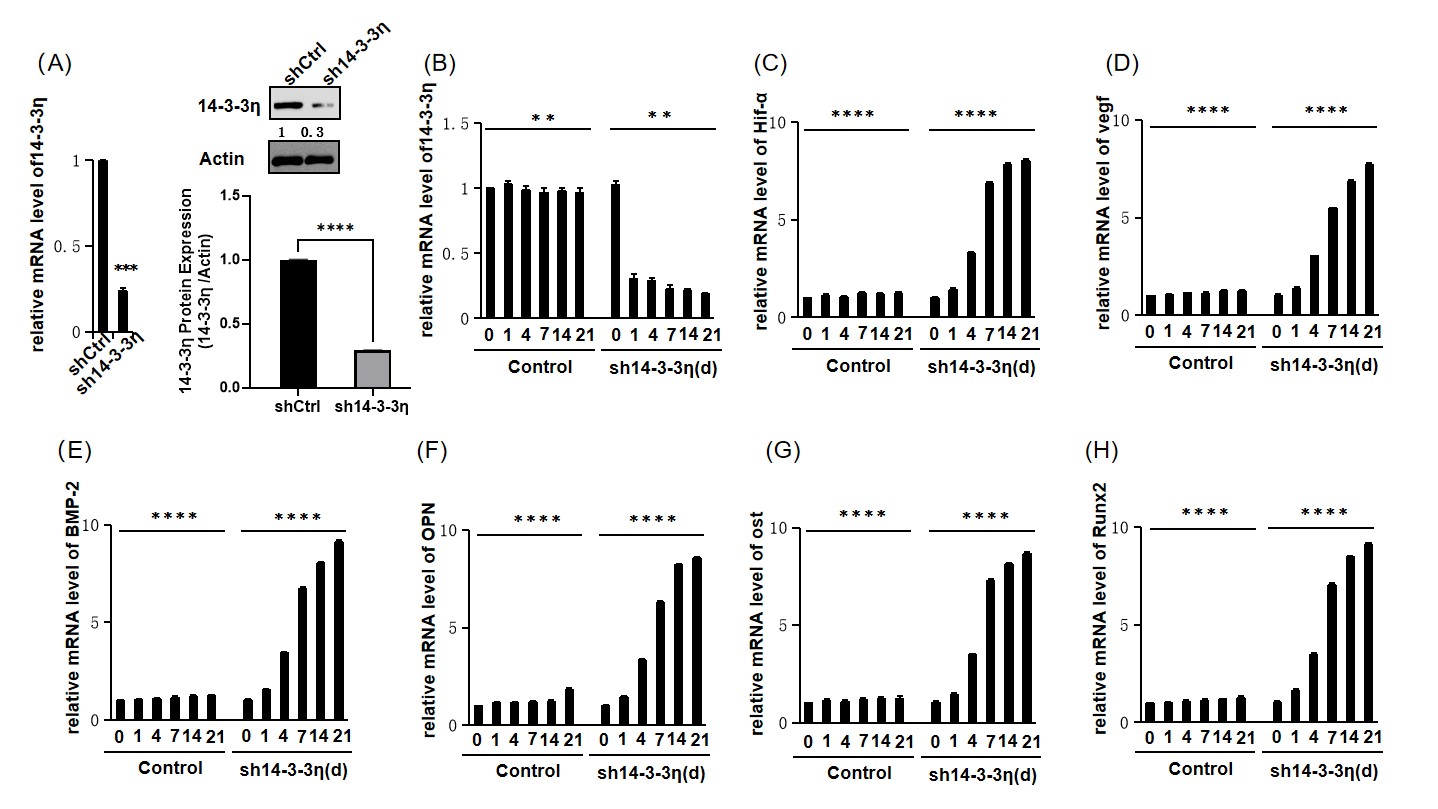


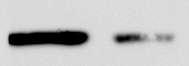
FIG3.14-3-3(1)


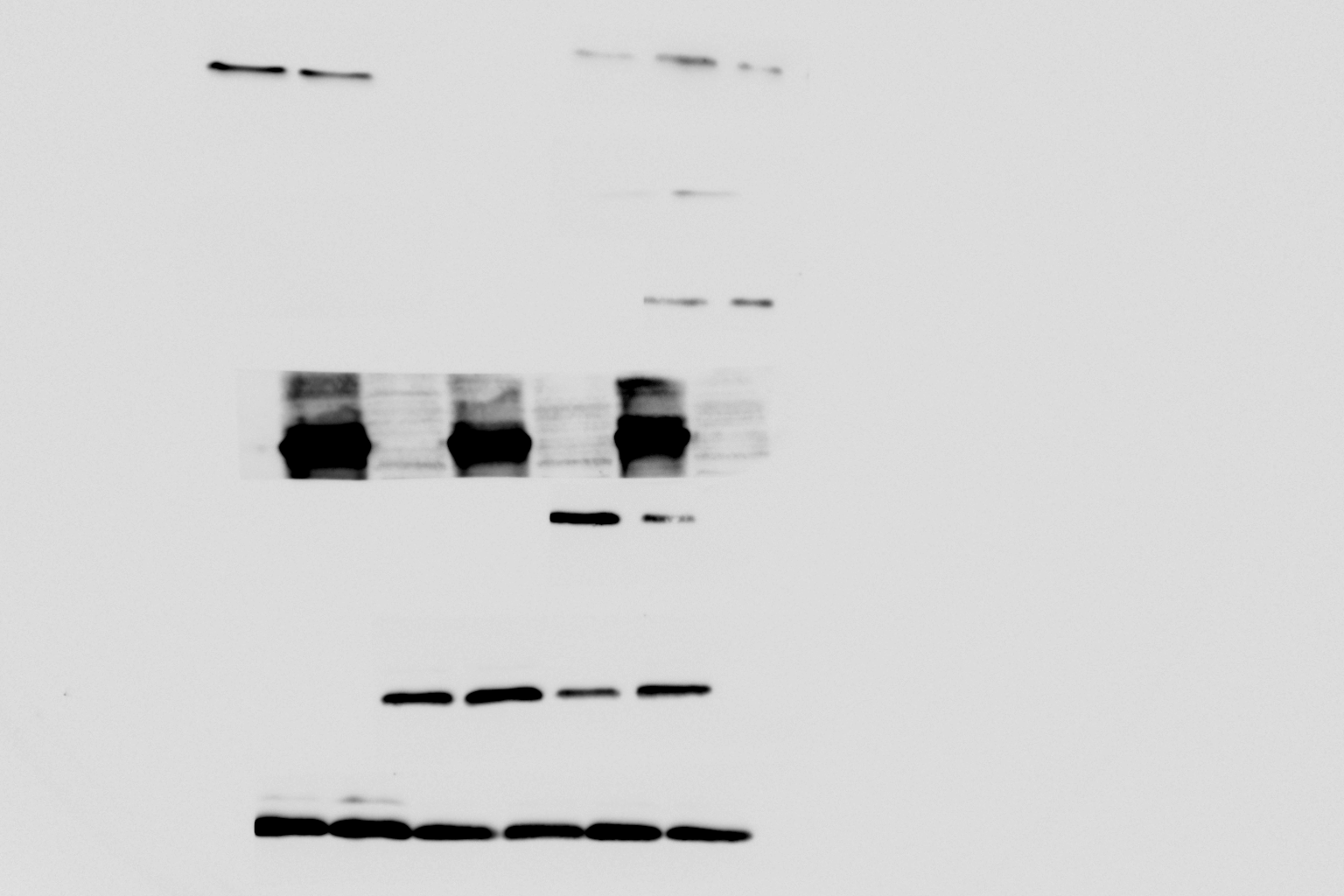
FIG3.14-3-3(2)


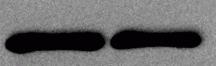
FIG3.actin（1）


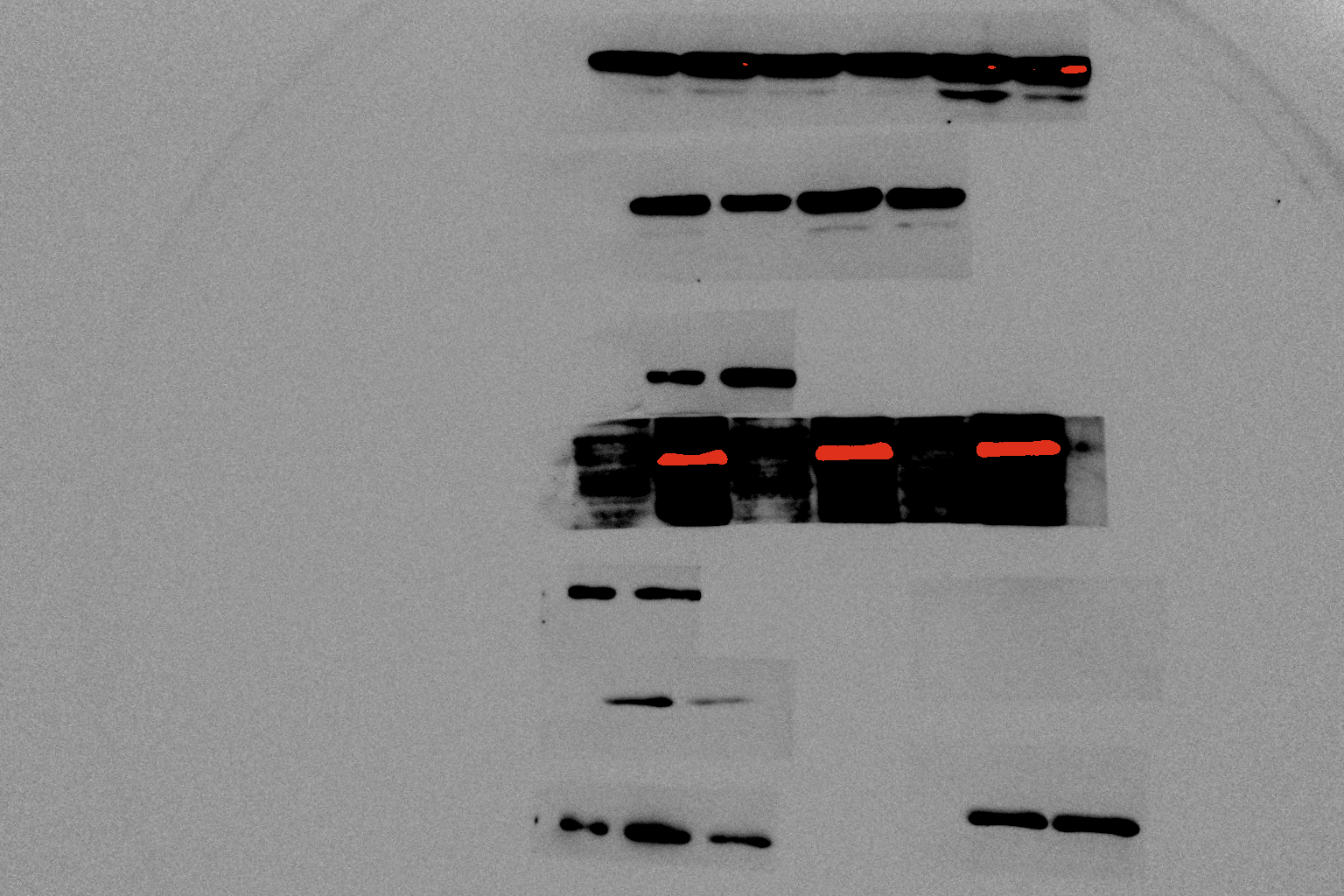
FIG3.actin（2）

Fig4：


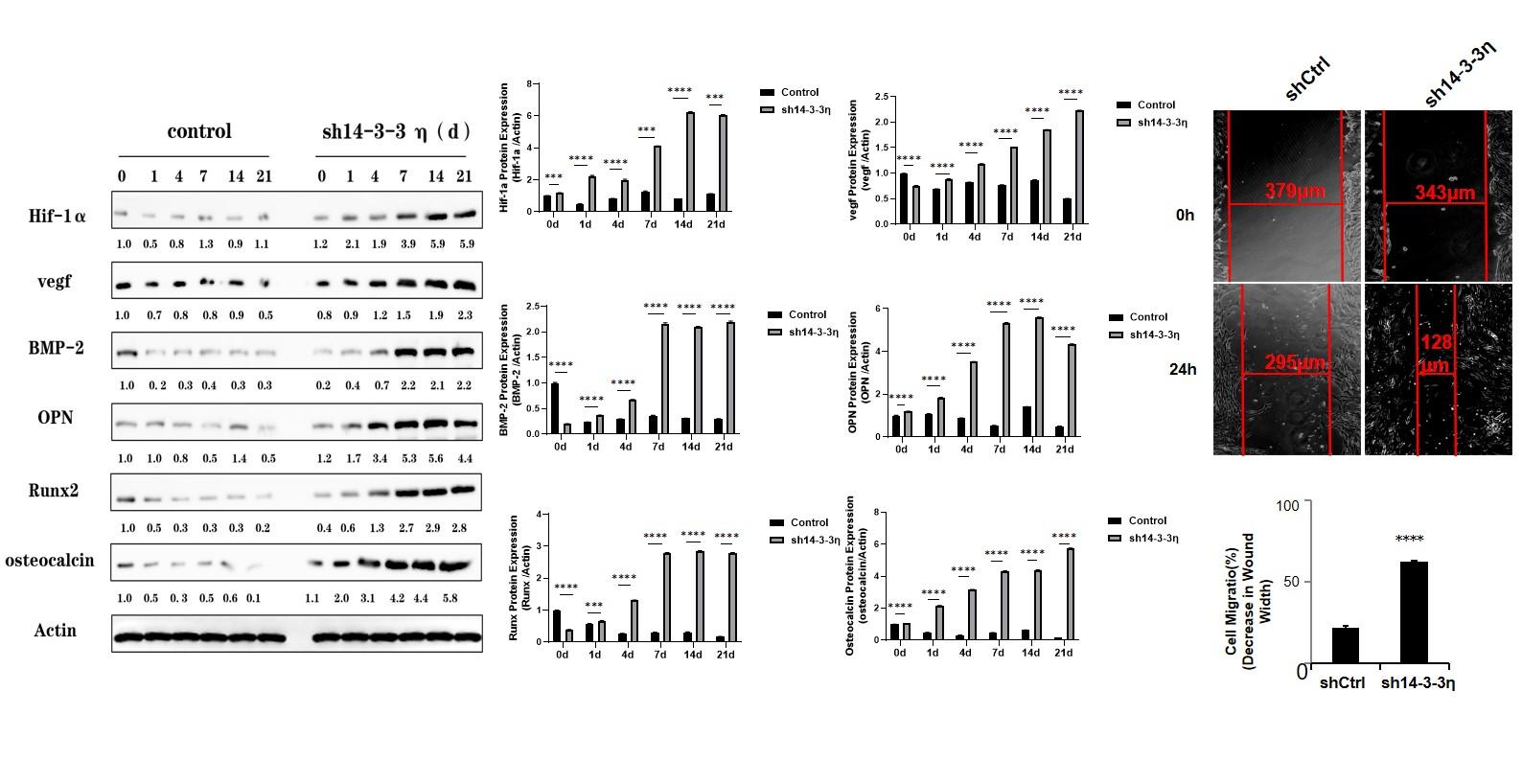


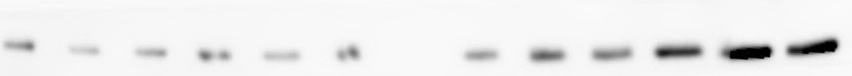
FIG4.hif-1


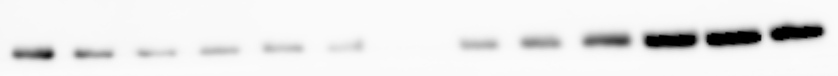
FIG4.runx2


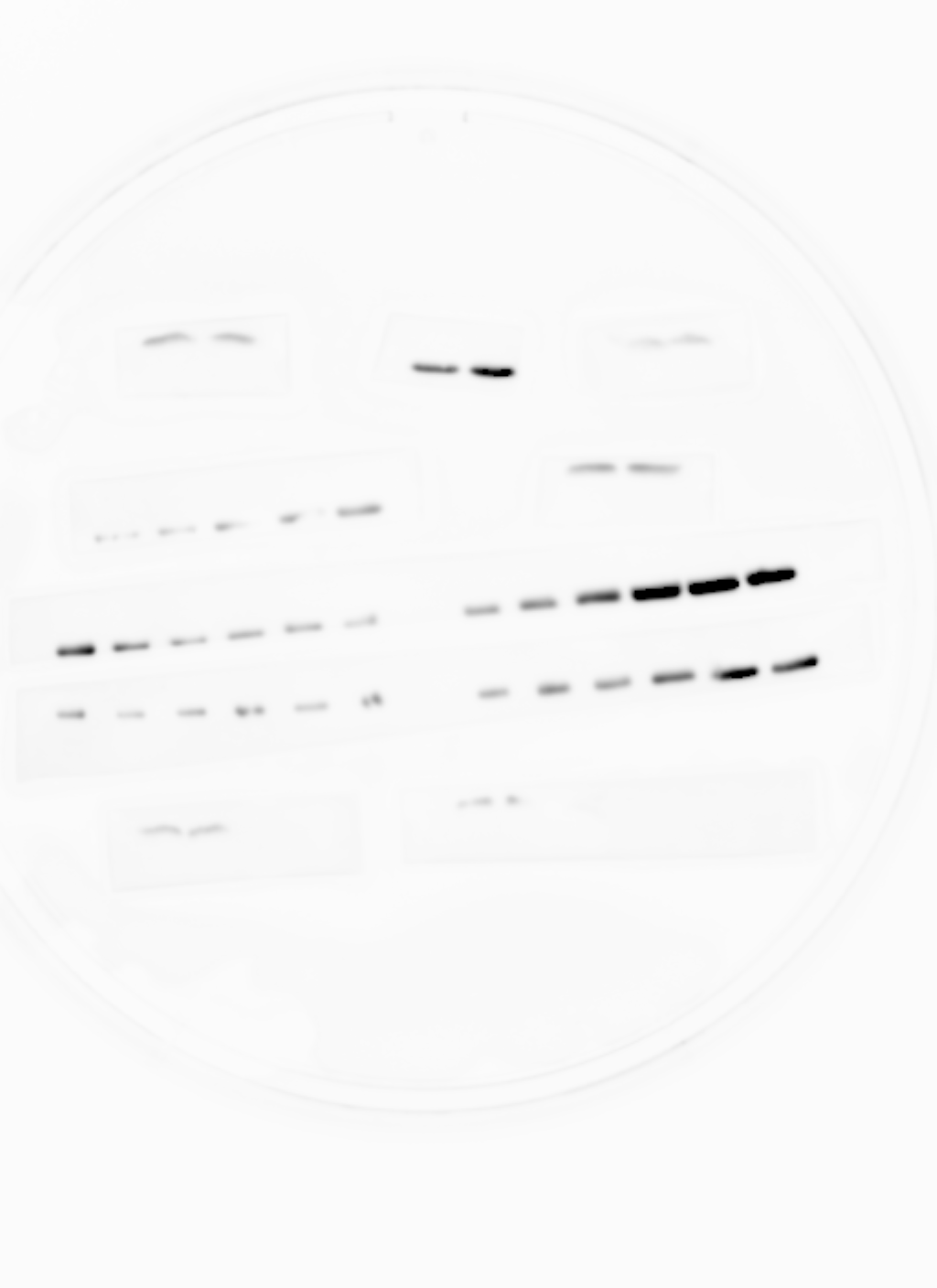
FIG4.HIF-1,RunX2


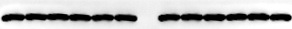
FIG4.Actin


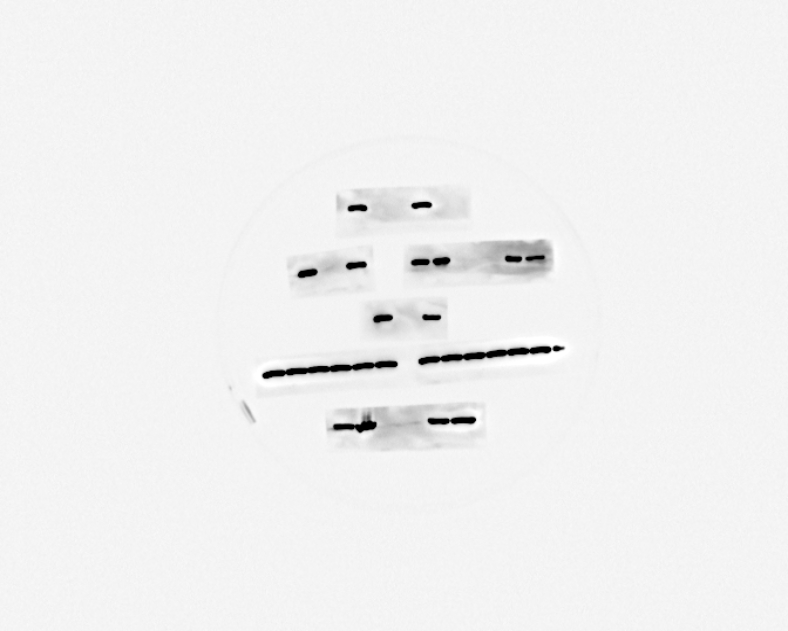
FIG4.Actin（2）


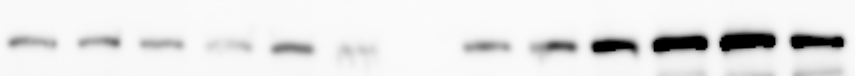
FIG4.OPN


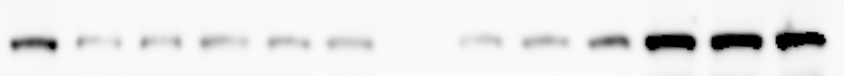
FIG4.BMP-2


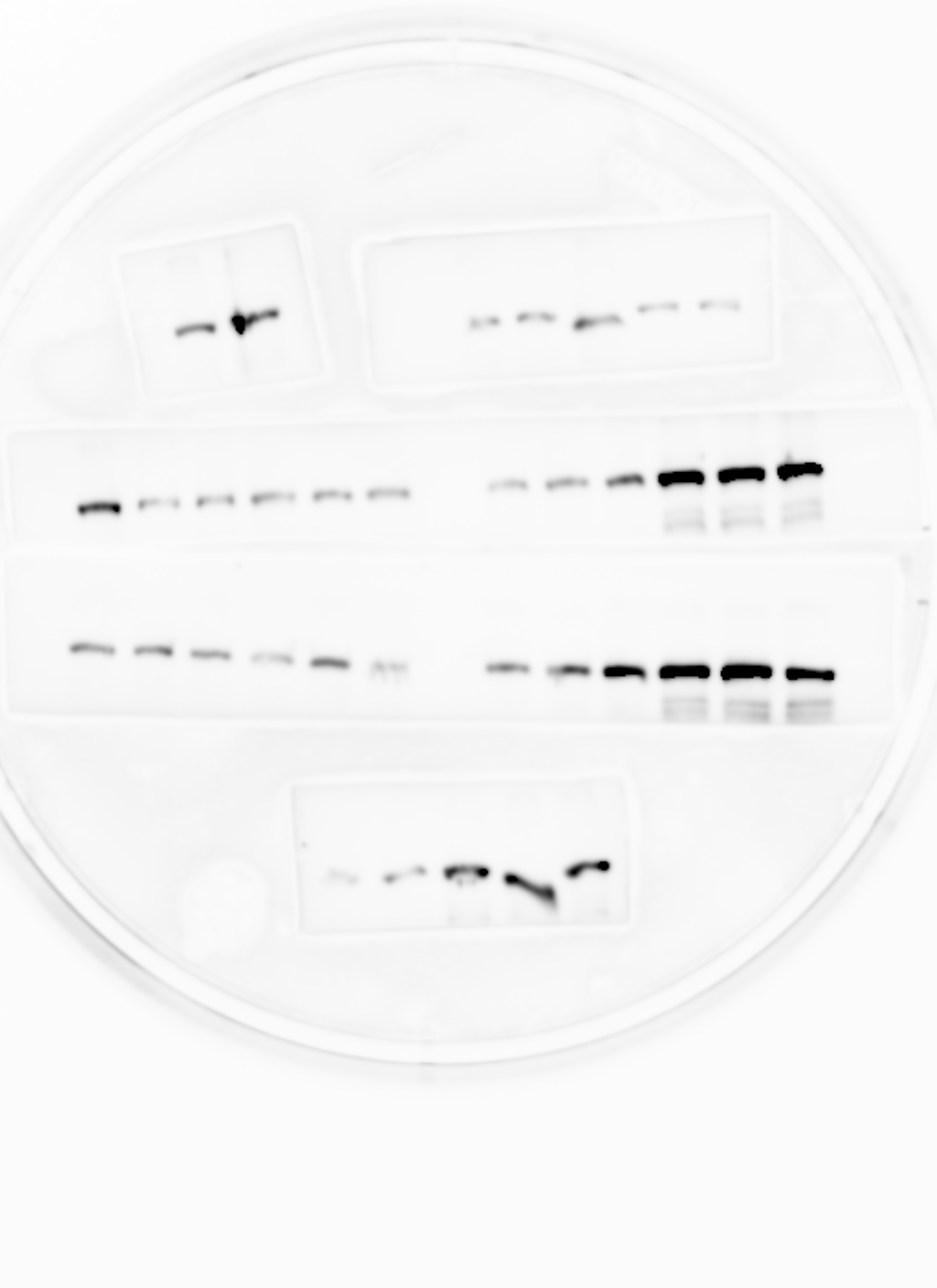
FIG4.BMP-2,OPN


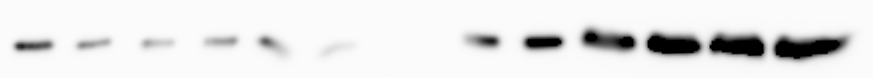
FIG4.osteocalcin


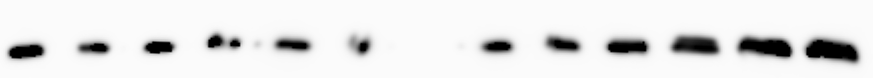
FIG4.vegf


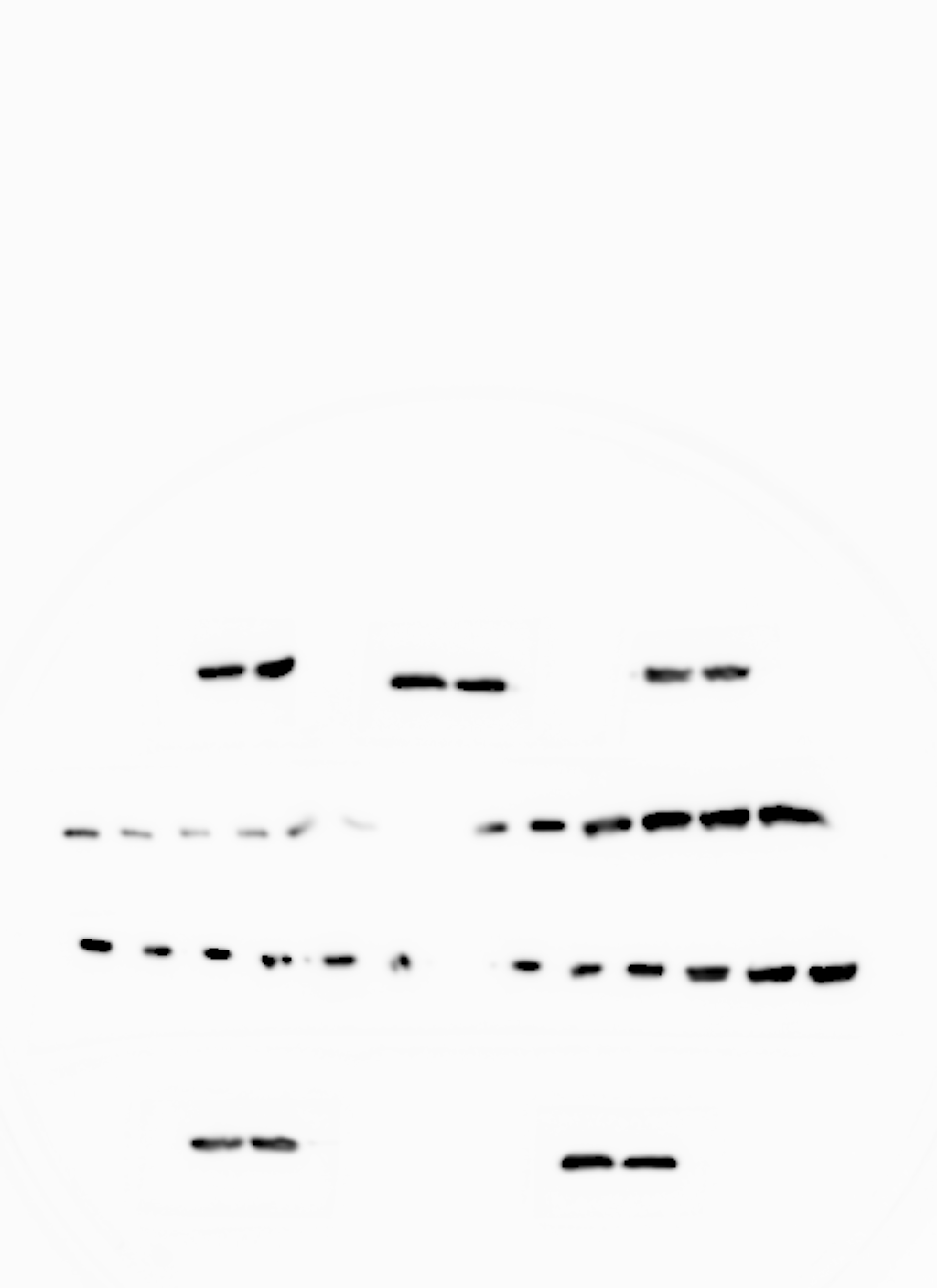
FIG4.osteocalcin，vegf

FIG5:


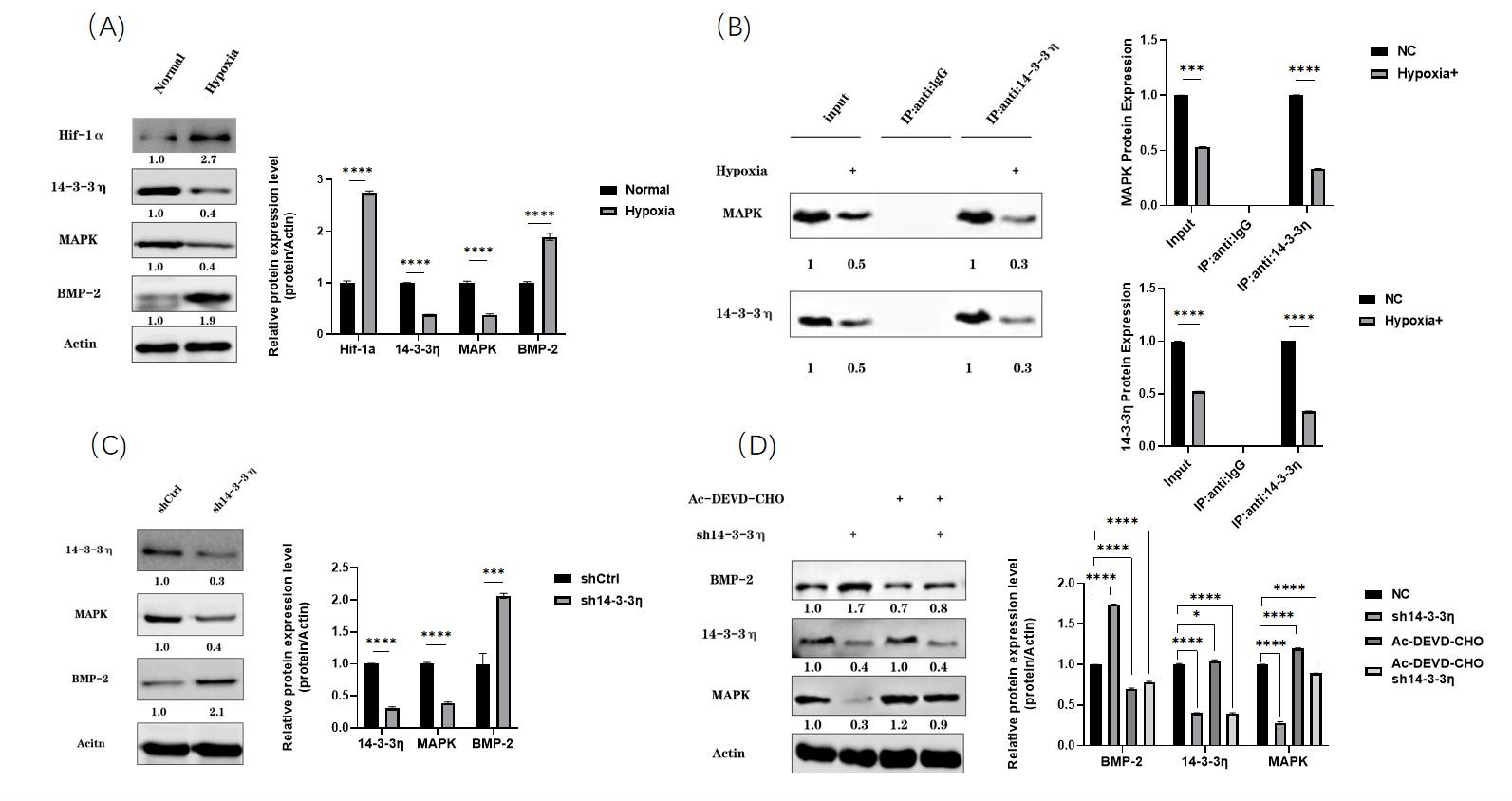


(A)



FIG5(A) Hif-1(1)



FIG5(A) Hif-1(2)



FIG5(A) 14-3-3（1）



FIG5(A) 14-3-3（2）



FIG5(A) BMP-2（1）



FIG5(A) BMP-2（2）



FIG5(A) Actin(1）



FIG5(A) Actin(2）

(B)


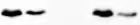
FIG5(B).MAPK


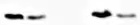
FIG5(B).14-3-3


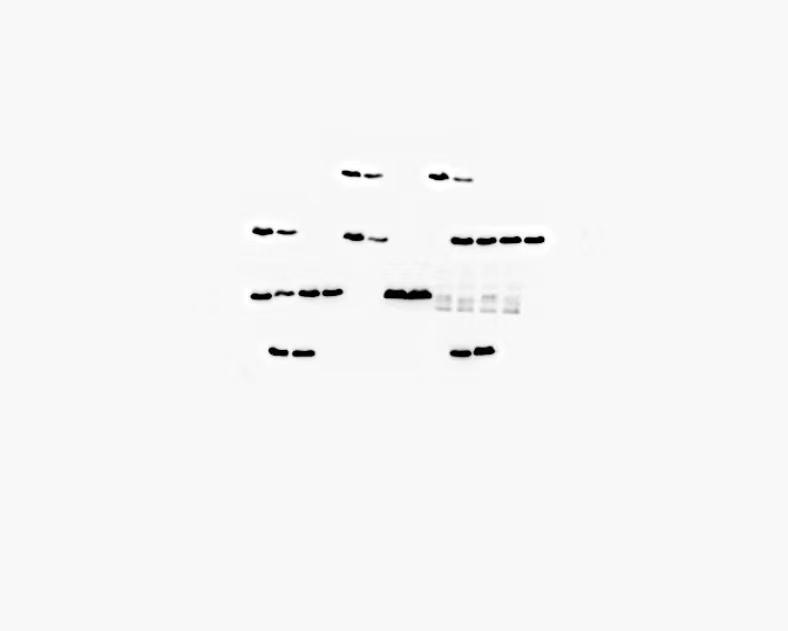
FIG5(B).14-3-3,MAPK

1. :



FIG5(C).14-3-3(1)



FIG5(C).14-3-3(2)



FIG5(C).BMP-2(1)



FIG5(C).BMP-2(2)



FIG5(C).Actin



FIG5(C)MAPK



FIG5(C).Actin &MAPK

(D):


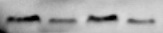
FIG5(D).14-3-3(1)


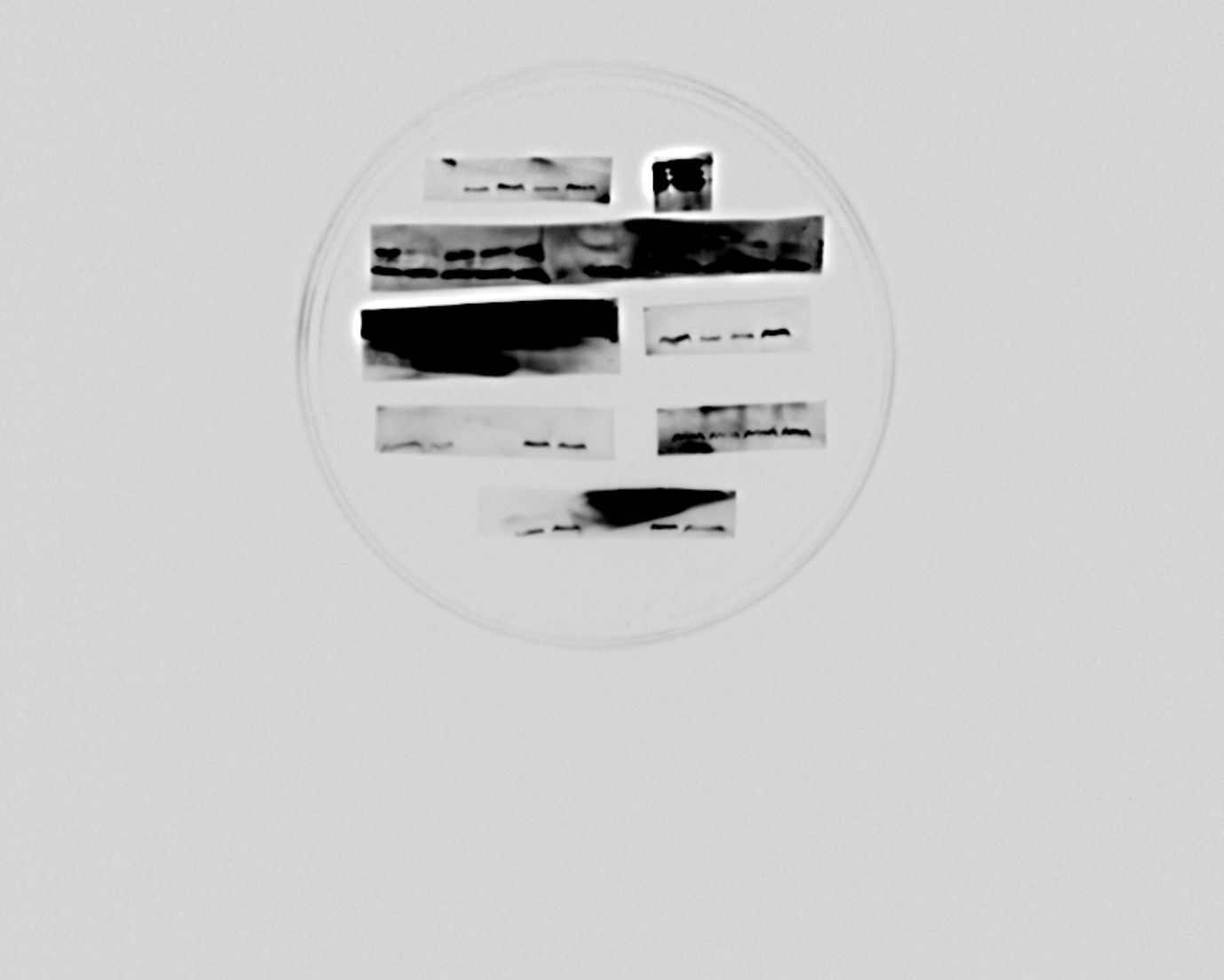
FIG5(D).14-3-3(2)


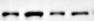
FIG5(D).BMP-2(1)


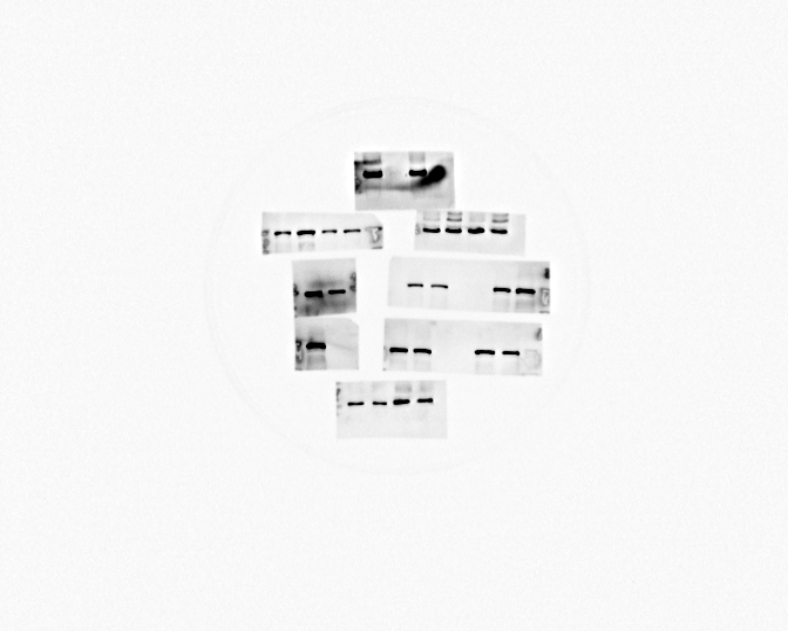
FIG5(D).BMP-2(2)


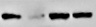
FIG5(D).MAPK(1)


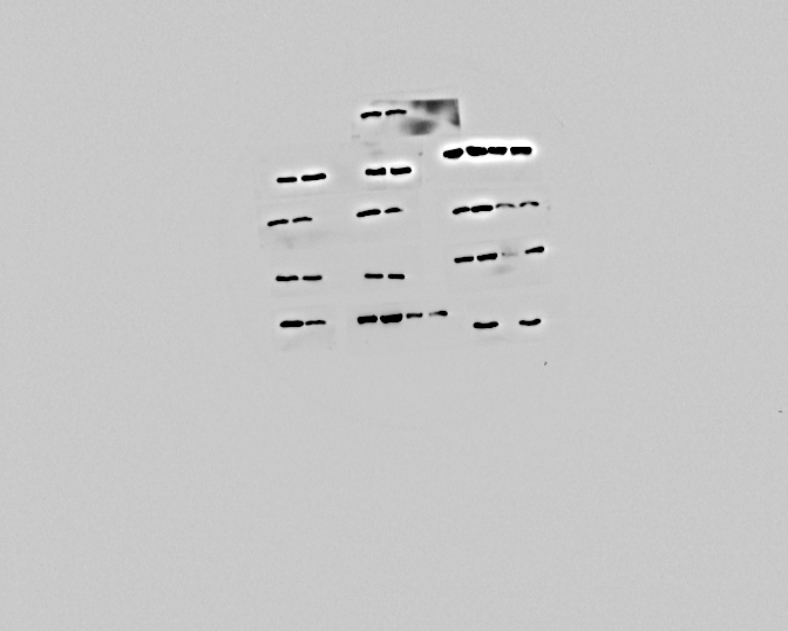
FIG5(D).MAPK(2)

FIG6:
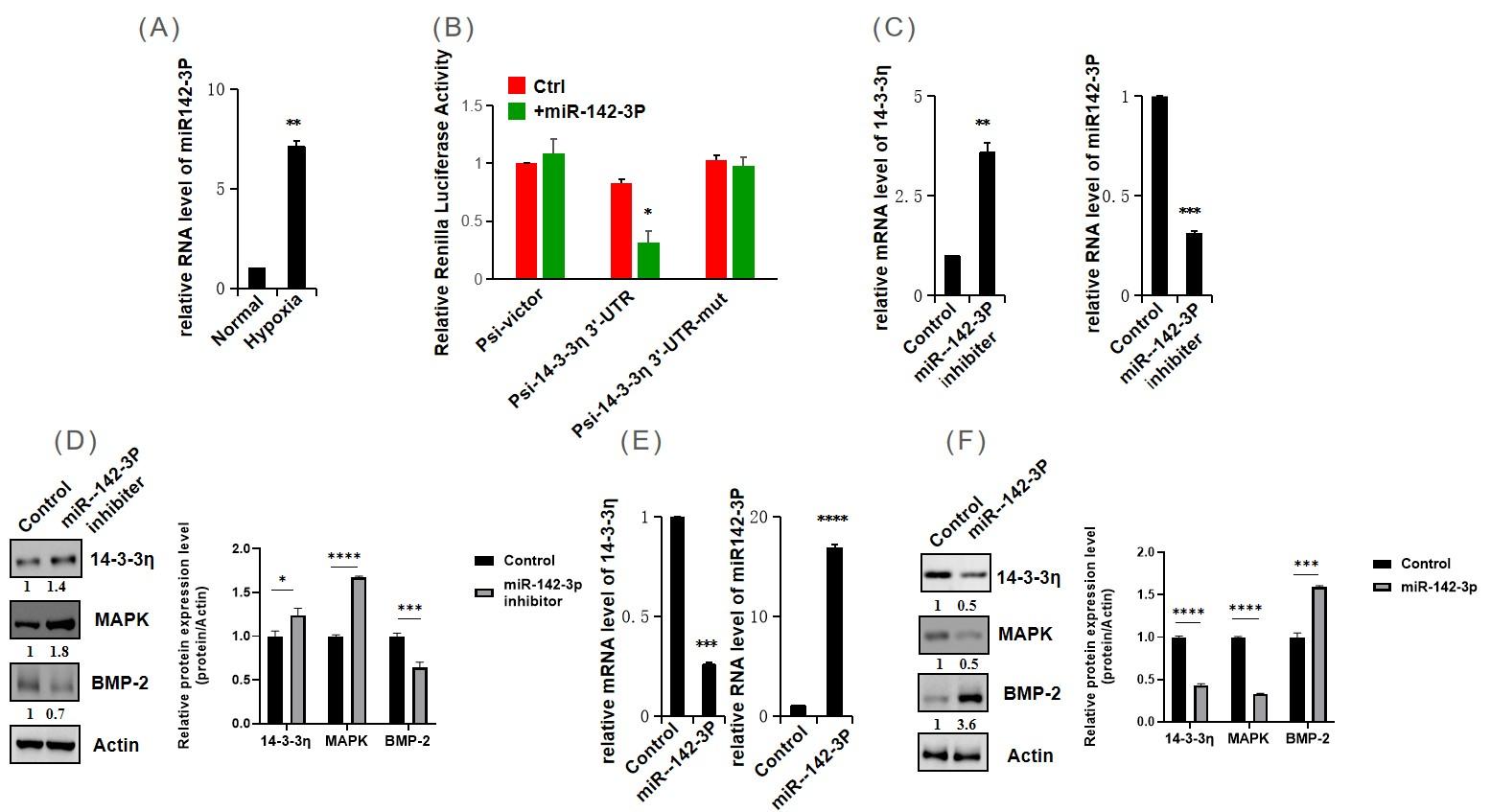


(D):



FIG6(D).14-3-3



FIG6(D).BMP-2



FIG6(D)14-3-3&BMP-2



FIG6(D).MAPK(1)



FIG6(D).MAPK(2)



FIG6(D). actin.(1)



FIG6(D). actin.(2)

(F):



FIG6(F).14-3-3(1)



FIG6(F).14-3-3(2)



FIG6(F).MAPK(1)



FIG6(F).MAPK(2)



FIG6(F).BMP(1)



FIG6(F).BMP(2)



FIG6(F).ACTIN(1)



FIG6(F).ACTIN(2)
