# Supplementary material for: Mir-142-3P regulates MAPK protein family by inhibiting 14-3-3η to enhance bone marrow mesenchymal stem cells osteogenesis
Source: Sci Rep. 2023 Dec 21;13:22862. doi: 10.1038/s41598-023-48950-4 (PMC10739902; doi:10.1038/s41598-023-48950-4)
Supplement: Supplementary file 2 — Supplementary Table 1. [file 41598_2023_48950_MOESM2_ESM.docx]

Supplementary Table 1. The primer information

|  | Forward Primer(5’- 3’) | Reverse Primer(5'-3’) |
| --- | --- | --- |
| B-actin | GTGGGGCGCCCCAGGCACCA | CTCCTTAATGTCACGCACGATTTC |
| 14-3-3 η | CTAGCGAGCCAGCGGTGTGA | CTGTCTCCAGCTCCTTCTCAATCTTCTCCC |
| miR-142-3p | GTCGTATCCAGTGCGTGT | ATTGCACTGGATACGACTCCAT |
| MAPK3 | CTTGACCTGCTAGACCGGAT | TCCAGCTCCATGTCGAAAGT |
| Hif-α | GACTATAGCTCCGGAGAATGC | TCGTATCTGGTCAGCTATGG |
| VEGF | GCGAGGCAGCTTGAGTTAAA | TCAGGCTTTCCTGGTGAGAG |
| BMP-2 | GTGACCCACTTGGAGGAGAA | CATCGTGGCCAAACGTTACT |
| OPN | AGTTCTGAGGAAAAGCAGC | CCCCTACCGGAACATACG |
| OST | GAGGGCAGCGAGGTAGTGAAG | GATGTGGTCAGCCAACTCGTCA |
| RUNX2 | GAGCCGGGTCATCTGAAGTA | TTTGCCATGTGGTTGTCAGG |
